# The Study of the Reliability of Complex Components during the Electromigration Process

**DOI:** 10.3390/mi14030499

**Published:** 2023-02-21

**Authors:** Hao Cui, Wenchao Tian, Yiming Zhang, Zhiqiang Chen

**Affiliations:** School of Electro-Mechanical Engineering, Xidian University, Xi’an 710000, China

**Keywords:** electromigration, reliability, life prediction, void formation, MTTF

## Abstract

With the increasing number of inputs and outputs, and the decreasing interconnection spacing, electrical interconnection failures caused by electromigration (EM) have attracted more and more attention. The electromigration reliability and failure mechanism of complex components were studied in this paper. The failure mechanism and reliability of complex components during the electromigration process were studied through the simulation and the experiment, which can overcome the limitation of experimental measurement at a micro-scale. The simulation results indicated that the solder joint has obvious current crowding at the current inlet, which will significantly enhance the electromigration effect. Based on the atomic flux divergence method, the void formation of solder joints can be effectively predicted, and life prediction can be more accurate than Black’s equation. Experimental results indicated that the resistance of the daisy chain could be significantly increased with the process of void formation in the solder and corrosion of the leads. Moreover, the growth of intermetallic compounds can be obviously promoted under current stress. The main composition of the intermetallic compounds changes from almost entirely Cu_5_Sn_6_ to Cu_5_Sn_6_ and Cu_3_Sn; the cracks can be detected at the Cu_3_Sn layer. Specifically, the mean time to failure is 1065 h under 1.4 A current and 125 °C based on IPC-9701A guidelines.

## 1. Introduction

Growing demand for emerging technology such as 5G, IoT, VR, cloud computing and metaverse has motivated the microelectronic industries to pursue device integration and advanced packaging technology because it enables devices with far higher interconnect density, higher power and smaller form factors which are ideal for the application of the complex functions [1,2]. The concerns are mostly related to the failure of the solder joint under high current stress. High-density current in metal interconnection structures will drive metal atoms to produce directional migration, which will cause mass transport, creating voids or hills in the structure [3,4]. The increased resistance, even a broken circuit caused by electromigration, could lead to severe reliability problems. While the technology has progressed significantly in recent years thanks to extensive developmental efforts, related studies have identified a few areas of concern that are critical challenges [5,6,7].

Many factors affect the IMC growth during the electromigration process. For instance, Rong studied the IMC growth behavior of solder joints with Cu/Sn/Cu structure and found that IMC showed a stronger resistance against failure under electromigration than solder [8]. Wang et al. reported that the solder composition had no apparent effects on IMC growth. The growing rate of IMC at the anode interface was far faster than that at the cathode interface [9]. Jin et al. conducted that Cu atoms were the dominant diffusion species that migrated from the cathode to the anode during EM. When IMC thickness decreased, the chemical potential increased and enhanced IMC growth [10]. Lin concluded that void formation was the most important failure mode during the EM test. The EM failures of micro bumps were observed through 3D X-rays, and the evolution of the voids can be checked without damaging the sample in the current study [11]. Abdelaziz reported that real-time viewing could well improve the understanding of electromigration in solder joints. Factors such as IMC, hillock formation, and grain rotation could be better understood through in situ imaging [12]. Qin analyzed the influence of the microstructure inhomogeneity of the solder matrix on the magnitude and distribution of the current density and temperature gradient in a microscale Cu/Sn58Bi/Cu solder. He pointed out that the distribution of the current density and temperature gradient in the Sn58Bi solder matrix under current stress can induce a severe crowding effect [13]. Xu conducted that the anode received a large number of Cu atoms from the cathode, and a large amount of IMC accumulated with the current stressing at 1.5 × 10^4^ A/cm^2^ at 125 °C, which mainly consisted of Cu_6_Sn_5_ and Cu_3_Sn [14]. Meanwhile, the orientation of the Sn grain had a considerable effect on the diffusion of Cu and electromigration damage. Madanipour et al. studied the extreme sensitivity of the microstructure evolution in the micro-solder joint. He found that the EM failure by void growth is in kinetic competition with the IMC growth rate, meaning that the joint achieves the condition of EM failure immunity when IMC growth outcompetes the voiding to convert the joint to the IMC phase fully [15]. Mao et al. studied the soldered joint’s microstructure evolution and mechanical properties with solid-liquid electromigration to determine the best bonding conditions. The addition of Ni foam enhanced the average tensile strength of solder joints. The highest tensile strength of the joints was 76.89 MPa when bonded at 0 A/cm^2^ current density compared with that 21.65 MPa at 6.67 × 10^2^ A/cm^2^ current density for eight hours [16].

The research on the influencing factors of electromigration has always been a hot topic for relevant scholars, including temperature gradient, stress gradient and current density gradient [17,18,19]. Basasran et al. predicted the time to failure for the electromigration process by a damage mechanics formulation. The void formation was not considered, unfortunately [20]. Park et al. presented a preliminary model based on the multiphase-field formalism which was used to simulate void formation process under the effect of electromigration [21]. However, it only applied to simple two-dimensional structures, not complex packaging structures. In the paper, we introduced the multi-physical field finite element method to estimate the electromigration process.

This paper studied the failure mechanism and reliability of complex components during the electromigration process through the simulation and the test experiment. Firstly, the electric and temperature fields were extracted through electrothermal coupling analysis with finite element software. Then, the solder joint void initial location and formation process during the electromigration process was simulated based on element birth and death technology. A corresponding experiment was designed and tested to observe cracks and corrosion during the electromigration process. Life predictions of complex components were based on the atomic flux divergence method and Black’s equation, respectively. Meanwhile, IPC-9701A guidelines were followed for the experimental method, including preparing the daisy chain test structure and definition of failure. Additionally, real-time viewing was used to complement the mean time to failure.

## 2. Simulation and Experimental Procedures

### 2.1. Simulation Approaches

**Figure 1:**
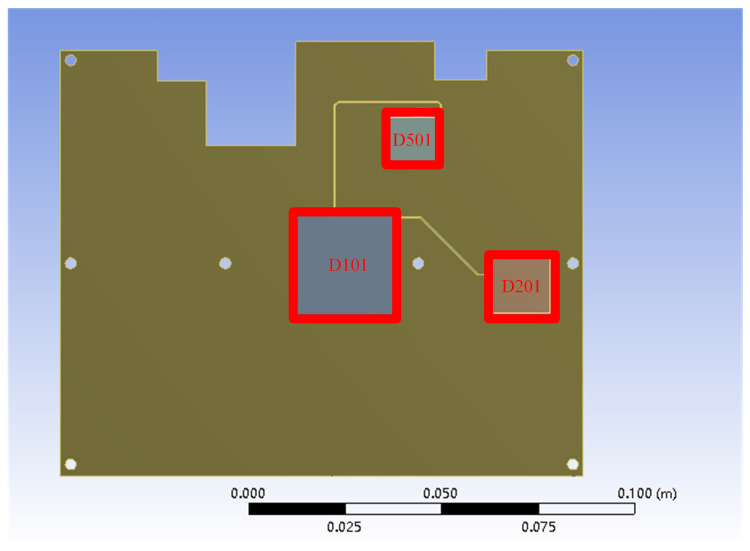
Structure diagram of the simulation model.

The closer the constructed 3D model is to the real physical PCB, the more accurate the simulation results. However, it is necessary to simplify the actual model and ignore some physical details to improve the simulation efficiency. Compared with the test samples, simulation model on one daisy chain including two BGA devices D101 and D201, and one QFP device D501 was used in the coupled analysis in Figure 1. The solder shape was simulated by surface evolver software based on the minimum energy principle and then exported to ANSYS finite element simulation software. Consistent with the following experimental conditions, the temperature constraint was imposed to 125 °C, and the thermal convection constraint was imposed to 1000 w/m.k considering the temperature control system by an oil bath. The positive pole was selected as the current application terminal (Current = 1.4 A), and the negative pole was the voltage application terminal (Voltage = 0 V). Meanwhile, element birth and death were used in the ANSYS workbench to set the failure elements and simulate the electromigration process. The material properties and packaging structure parameters were listed in Table 1 and Table 2, respectively.

### 2.2. Electromigration Test

In order to enhance the testing accuracy, typically 32 samples were tested in an oil bath system at 125 °C with current density stress loads of 1.4 A designed for effective joule heat dissipation and temperature uniformity across the samples. Meanwhile, a daisy chain circuit, including two BGA devices and one QFP device was used to supply stable current density and be constantly electrical monitored by an event detector and a data logger. The data loggers were used to detect and record resistance change. Based on industry-standard IPC-9701A, failure definition could be described as a 20% nominal resistance increase in a daisy chain circuit. Similarly, mean fatigue life could be defined as the amount of time at which one-half of a given sample has failed, so the failure time of the 16th PCB was regarded as failure life.

**Figure 2:**
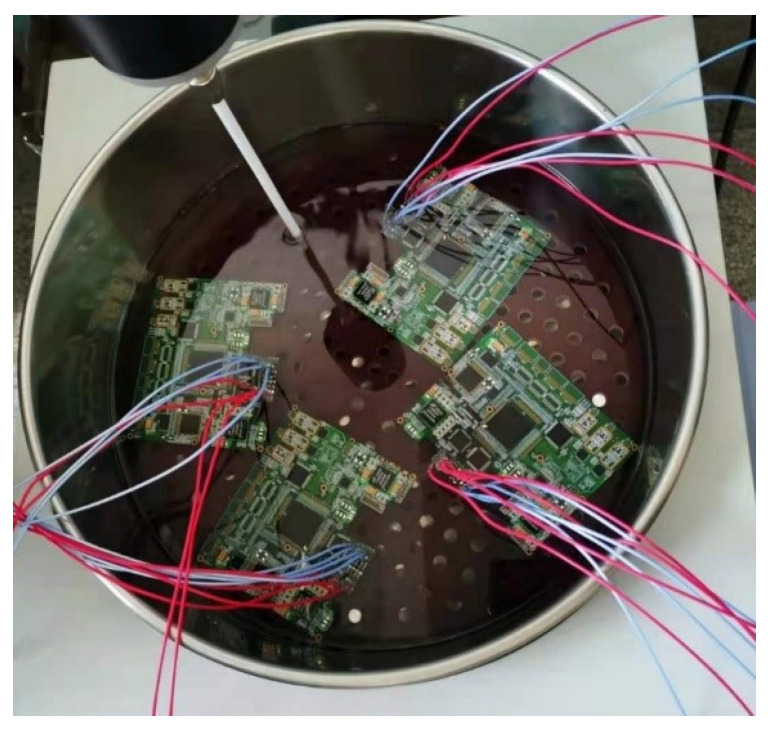
Samples in the oil bath heater.

**Figure 3:**
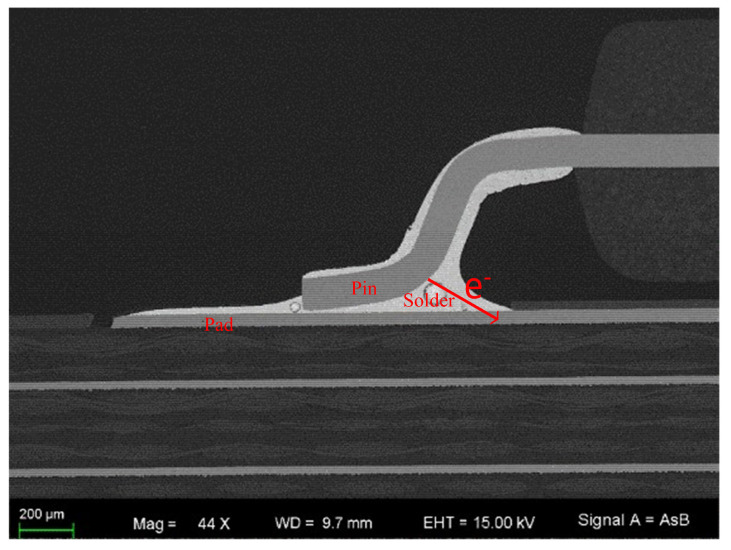
An SEM image of a QFP.

**Figure 4:**
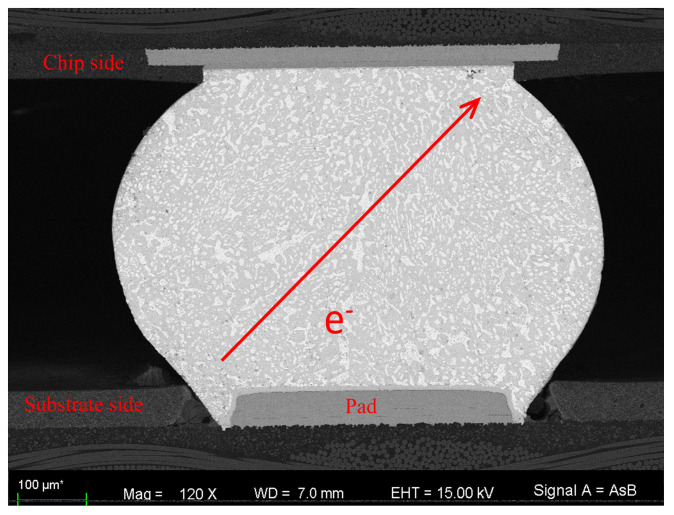
An SEM image of a BGA.

The prepared samples were then subjected to high-resolution microscopy using an SEM with energy dispersive spectroscopy (EDS) and electron backscatter diffraction (EBSD) systems. The IMC thickness was measured by scanning electron microscopy (SEM) at 5000× magnification. The IMC layer’s total area was measured using a computer drawing tool. Area divided by length was considered the average value of the IMC thickness used for analysis because of the non-uniform distribution of the IMC layer. The samples in the oil bath heater are shown in Figure 2. As shown in Figure 3 and Figure 4, it can be seen that the bonding quality was good without voids, cracks and other defects.

## 3. Results and Discussion

### 3.1. Thermo-Electric Coupling Analysis

**Figure 5:**
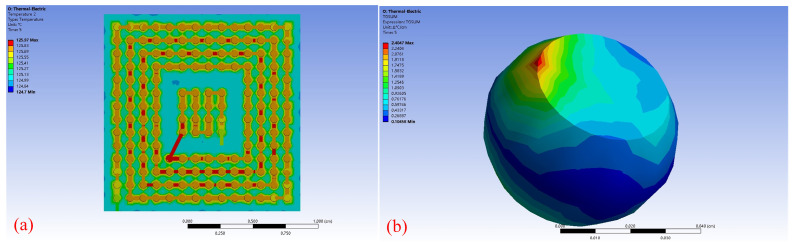
Simulation results of the temperature (**a**) Temperature distribution in D501 (**b**) Temperature gradient in solder joint.

Figure 5a shows the simulation temperature at the beginning of the tests at 125 °C and 1.4 A of D501. Due to the effect of oil bath temperature control, the BGA device would have a certain temperature rise due to the current joule thermal effect, which would raise the temperature from 125 °C to 125.97 °C. However, due to the small size of the solder joint, the temperature varied little, and the maximum temperature gradient was 2.4047 °C/cm at the current inlet as shown in Figure 5b.

The formation of the void is mainly caused by the atomic divergence, and the atomic migration usually occurs firstly at the position with the largest atomic divergence. The atomic divergence ($div(J_{em})$) caused by the current is given by the following equation [22]:

(1) $$\;div(J_{em})=\left(\frac{E_{a}}{KT^{2}}\right.-\frac{1}{T}+\alpha \left.\frac{\rho_{0}}{\rho }\right)J_{em}\cdot \nabla T$$

where *K* is Boltzmann’s constant, *T* is temperature, *ρ* is resistivity, *E_a_* is the activation energy. $\rho_{0}$ is initial resistivity. α is temperature coefficient of resistivity, $J_{em}$ is the atomic flux due to the electromigration, ∇T is the temperature gradient.

Furthermore, the atomic divergence caused by the temperature gradient ($\mathrm{div}\left(J_{tm}\right)$) and stress ($\mathrm{div}\left(J_{sm}\right)$) could be given by the following equation, respectively.

(2) $$\mathrm{div}\left(J_{tm}\right)=-\left(\frac{E_{a}}{KT^{2}}-\frac{2}{T}\right)J_{tm}\cdot \nabla T-\frac{CD}{KT^{2}}Q^{2}\nabla^{2}T$$

(3) $$\mathrm{div}\left(J_{sm}\right)=\left(\frac{E_{A}}{KT^{2}}-\frac{1}{T}\right)J_{sm}\cdot \nabla T-\frac{\Omega D}{KT}\nabla^{2}\sigma_{H}$$

where *D* is the diffusion coefficient, *C* is the atomic concentration, *Q* is the molar heat energy during migration, $J_{tm}$ is the atomic flux due to the thermomigration, $J_{sm}$ is the atomic flux due to stress migration, Ω is atomic volume and $\sigma_{H}$ is the average value of stress in three directions of x, y, z.

The effect of current density, the temperature gradient caused by Joule heating, and the stress caused by the mismatch in the material properties are all considered. The total mass flux divergence can be calculated as the following equation:

(4) $$\;\mathrm{div}(J)=div(J_{em})+div(J_{tm})+div(J_{sm})$$

**Figure 6:**
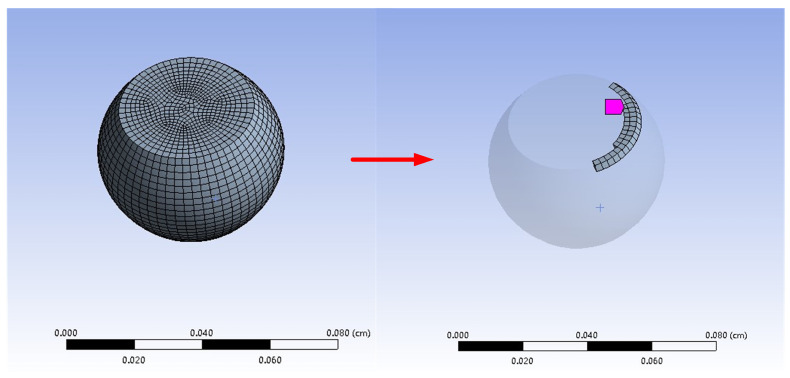
Simulation of void formation process in solder joints.

The steady-state analysis located the initial situation of the void and then determined the weakest part of the solder joint. The birth and death technology was used to kill the element with the largest total mass flux divergence and then the model was reconstructed to conduct thermal electrical coupling analysis. Figure 6 shows the simulation of the void formation process in solder joints in electromigration.

**Figure 7:**
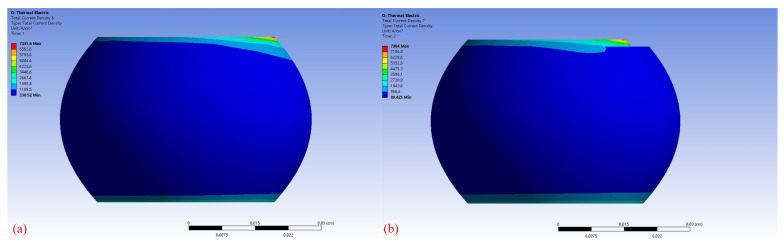
Simulation results of the current density distribution (**a**) The initial condition (**b**) The void volume reaches 5%.

Figure 7 shows the current density of BGA at different stages. In the initial condition, the maximum current density of the solder joint at the current inlet was 7341.6 A/cm^2^, which indicated that the current crowding effect was obvious. This current density at the entrance was more than ten times higher than the average current density. With the formation of voids, the maximum current density at the solder joint increased continuously. When the void volume reached 5%, the maximum current density increased to 7984 A/cm^2^ leading to an accelerating electromigration process. The current crowding location shifted from the solder joint corner to the void edges during the electromigration process.

### 3.2. Failure Time Calculation Based on Black’s Equation and Atomic Flux Divergence Method

In the microelectronic industry, mean-time-to-failure (MTTF) is used to evaluate reliability. The MTTF is defined as the average normal working time before failure, which is one of the key factors in evaluating reliability. Black’s equation of mean time to failure (MTTF) for electromigration has been given as [23]:

(5) $$MTTF=A\frac{1}{j^{n}}\exp\frac{Q}{KT}$$

where *A* is the constant, *Q* is the activation energy of atomic diffusion in electromigration. *K* is the Boltzmann constant; *T* is the absolute temperature, j is the current density, and n is the current density power factor.

To modify Black’s equation, current crowding and Joule heating effect should be considered. The modified Black’s equation is given as [24]:

(6) $$MTTF=A\frac{1}{{\left(\mathrm{cj}\right)}^{n}}\exp\left(\frac{Q}{K\left(T+\Delta T\right)}\right)$$

where ΔT is the temperature rise caused by current. C is the current density influence factor. The current density and temperature can be extracted through ansys workbench software. In Equation (6), if we keep both n and Q constant, we see that the effect of both c and ΔT is to reduce MTTF. Both of them should depend on the applied current density. The activation energy Q is 0.8, the current density power factor n is 1.8, the current density influence factor is 1, A is 0.4 by reference [25]. If we take the ΔT as 0.97 °C, the max current density 7.3 × 10^3^ A/cm^2^ at the initial state by simulation results, the expected *MTTF* values were calculated as 588 h based on Equation (6).

With the development of the void formation, a strong increase in the electrical resistance could be determined, indicating the electrical failure. The element with the largest total mass flux divergence migrates most easily.

When the ratio of the voids interface area to the voids-free interface area reaches 50%, it can be judged that the solder joint has failed [26]. The voids-free interface has about 720 elements, the time consumed is the lifetime of the solder joint when 360 elements migrated. The electromigration failure time is inversely proportional to the atomic flux divergence. We can suppose that the current atomic concentration of a unit decreases to 10%, the atoms have been migrated, and the element should be killed in the finite element analysis. The mean-time-to-failure can be given as the following equation:

(7) $$\begin{array}{l} t_{i}=\frac{\ln(10)}{div(J)}\\ MTTF=\sum_{i=1}^{360}\frac{\ln(10)}{div(J)}=\sum_{i=1}^{360}t_{i} \end{array}$$

The simulation process could be divided into ten steps, where 30 elements with the largest total mass flux divergence were killed. Then, the total mass flux divergence was calculated again and determined the next steps of 30 elements deletion. Then the process was cycled until 360 elements were killed. The results are listed in Table 3. On the one hand, with the increase of steps, the max total mass flux divergence continued to increase, and the growth rate also increased. On the other hand, the average life of each element decreased with the increase in the void volume. The total failure time of solder joints could be calculated as 954.91 h based on Equation (7).

### 3.3. Failure Time Based on Experiment Results

**Figure 8:**
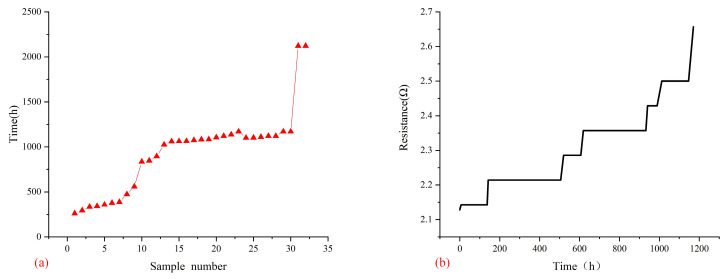
Results of electromigration experiment (**a**) Lifetime distribution of the test samples (**b**) The 16th sample of electrical resistance of daisy chain circuit as the function of time.

Figure 8a shows the 16th sample of electrical resistance of the daisy chain circuit tested at 125 °C at 1.4 A as a function of time. The lifetime distribution of the test sample, which could be seen as the first sample to fail, was about 250 h, considered infant mortality. The failure time of most devices was about 1000 h- 1065 h was the failure time of the 16th test sample, which was the mean time to failure time defined by the IPC-9701A standard. Figure 8b shows the 16th sample of electrical resistance of the daisy chain circuit tested at 125 °C at 1.4 A as a function of time. It can be seen that the resistance of the daisy chain increased gradually with time. The results showed that the increase process of its resistance was not a continuous process, and it often changed significantly in a few minutes or even seconds. The initial resistance of the daisy chain was 2.12 Ω; until the failure, its resistance changed to 2.65 Ω, while the recorded value before the failure happened was 2.5 Ω. That was due to the rapid propagation of cracks in a short time.

Table 4 shows the failure time results based on different methods. Both two methods had certain errors compared with the results of the experiment. The atomic flux divergence method could accurately predict solder joints void formation during the electromigration process and had a 10.33% error less than that 44.78% based on Black’s equation. The predicted failure time of the two methods was shorter than the experimental test because the simulation only considered the life of the dangerous location rather than the change of the whole daisy chain. On the other hand, the parameter in the Black’s equation given by reference may led to inaccuracy values. The empirical geometry-related parameter A can be estimated as 0.66 based on the experiment results in Equation (6). However, the method based on Black’s equation was simple and convenient, which was suitable for the initial product design.

**Figure 9:**
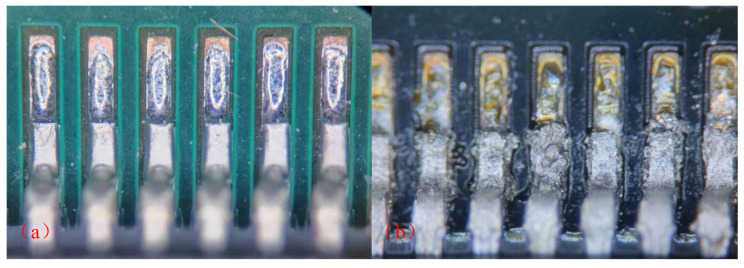
Enlarged view at D501 device pins (**a**) Before the electromigration test (**b**) After the electromigration test.

Moreover, as shown in Figure 9, significant changes could be observed at D501 device pins after the electromigration. The surface had a metallic luster and was smooth, without cracks and inclusions before the test by a microscope. However, severe electrochemical corrosion of pins had been observed under the microscope.

**Figure 10:**
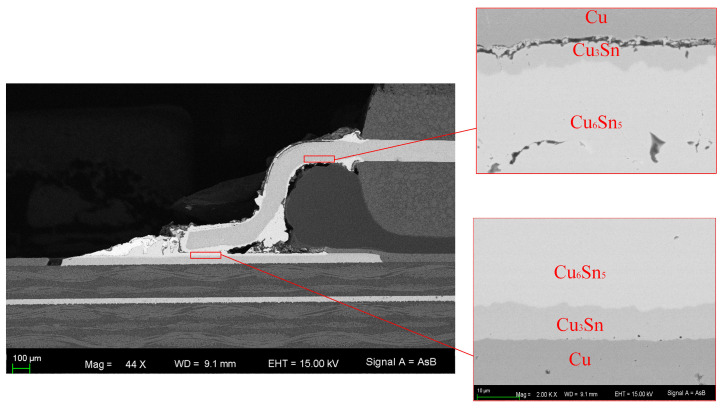
An SEM micrograph showing the QFP device pin after the electromigration test.

Figure 10 shows the QFP device pin after the electromigration test. There were obvious cavities and cracks at the pins due to the effect of electromigration. The morphology of the intermetallic compound layer Cu_3_Sn and Cu_6_Sn_5_ was clear and flat on the substrate side after the test. Cracks and Kirkendall voids were observed at the interface between Cu and Cu_3_Sn layers. Thus, the kinetics of IMC growth during the EM process should be further studied.

### 3.4. Kinetics of IMC Growth during the EM Process

During the EM test, the effect of changes in IMC thickness and void formation can be observed, which depends on the polarity at the cathode and anode. In fact, electromigration is not a completely independent process.

For example, current flow will inevitably produce Joule heat, and higher temperature will lead to instability of atoms, which will also accelerate the chemical reaction process. The atoms can be more easily freed from the bonds of surrounding atoms under the higher current, moving directionally from the cathode to the anode. Moreover, the cathode will form a tensile stress region due to the migration of atoms, and the anode will form a compressive stress region due to the migration of atoms which results in a baffling effect.

In the real situation, the growth of IMC depended mostly on Cu diffusion, which was taken as an example in this paper. Because the Sn atoms were quite limited, the free Cu atoms could be continuously provided by Cu substrates. The chemical potential was the driving force for IMC growth for aging.

Based on Fick’s law, the Cu flux due to potential chemical gradient could be expressed as [27]:

(8) $$J_{Chem}=D\frac{dC}{\mathrm{dx}}$$

where *D* is a constant of Cu diffusivity in IMC, *C* is atomic concentration of Cu and d*C*/dx is the gradient of Cu concentration.

Besides chemical potential, electromigration and back stress caused by electric current also played an important role in the IMC growth during the electromigration. The Cu flux due to electromigration-driven stress and back stress could be expressed as:

(9) $$J_{EM}=\frac{DC}{KT}Z^{*}e\rho j$$

(10) $$J_{\sigma }=\frac{DC}{KT}\Omega \frac{d\sigma }{\mathrm{dx}}$$

where *K* is the Boltzmann constant, *T* is the absolute temperature, Z* is the effective charge number, *e* is the electric charge quantity, *ρ* is the resistivity, j is the current density, Ω is the atomic volume, σis the hydrostatic stress, and dσ/dx is the stress gradient.

**Figure 11:**
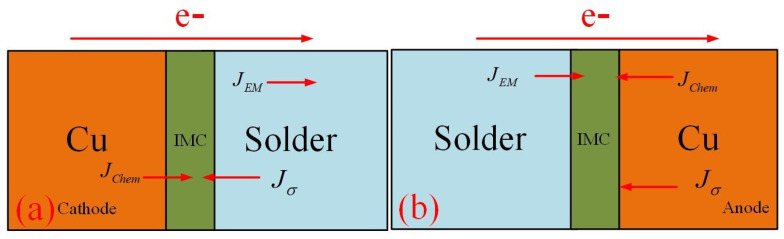
Diffusion models at the cathode and anode.

However, atoms moved in a directional direction under electric current, leading to IMC growth at the anode and cathode totally different. The diffusion models at the anode and cathode of the electromigration process are shown in Figure 11a,b, respectively. The accumulation of Cu atoms at the interface created compressive stress and built a stress gradient from the anode to the cathode.

At the anode, under the combined effect of chemical potential gradient, electromigration and back stress, the IMC thickness always increased with time. With increasing time, the thicker IMC reduced the gradient of Cu concentration which resulted in the growth speed of the IMC decreasing. At the cathode, electromigration could stunt IMC growth. As the current density changed, the IMC growth all came differently. The thickness of IMC reduced ($J_{EM}>J_{\sigma }+J_{Chem}$) in high current density and increased ($J_{EM}<J_{\sigma }+J_{Chem}$) in low current density.

**Figure 12:**
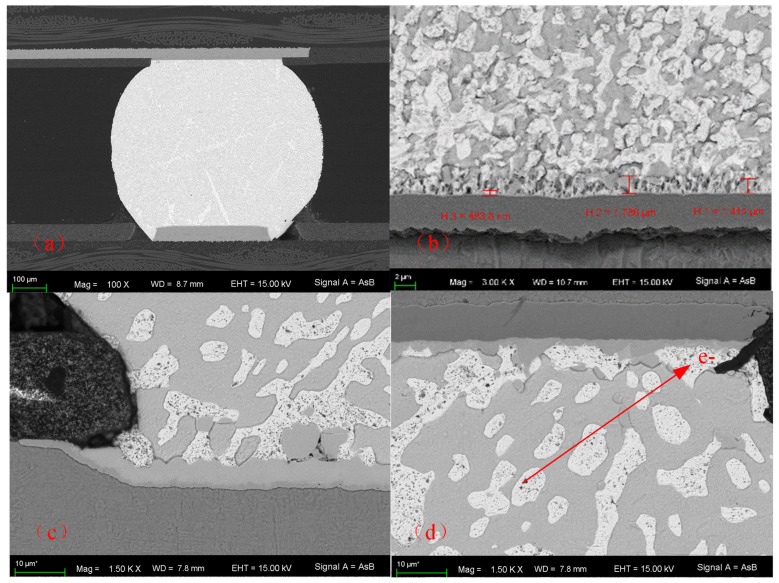
SEM image of BGA (**a**) Before electromigration experiment (**b**) SEM image of IMC (**c**) SEM image at the anode after the electromigration experiment (**d**) SEM image at the cathode after the electromigration experiment.

As shown in Figure 12a, the solder had good weld quality and no void could be detected. It was observed that the formation of a dual Cu–Sn IMC layer consisting of Cu_6_Sn_5_ phase exists between the solder and substrate in Figure 12b. The IMC became thicker and smoother at the anode as shown in Figure 12c. Moreover, the void could be clearly observed at the cathode after the electromigration experiment in Figure 12d. As a void propagates due to current, it could be expected that the current crowding location shifts from the solder joint corner to the void edges which are basically in agreement with the simulation results. Electrons were forced to flow around the void, leading to a higher current density and intensifying the current crowding effect.

## 4. Conclusions

In this study, 3D FE analyses and experiments were performed to evaluate the influence of the reliability of complex components under electromigration conditions. The following conclusions are drawn:

(1) The temperature rise and temperature gradient of the solder joint were very small under oil bath conditions. By simulation of current distribution in a solder joint, we confirmed that the existence of current crowding occurs at the top side. With the increasing void volume, the maximum current density increased leading to an accelerating electromigration process. The current crowding location shifted from the solder joint corner to the void edges during the electromigration process.

(2) The experiments have proven that electromigration affected the growth of the intermetallic compound at the anode and cathode of solder joints leading to a thicker and smoother IMC layer at the anode and void formation at the cathode. Similarly, the pin could be seriously electrochemical corroded, and cracks and Kirkendall voids at the interface between Cu and Cu_3_Sn layer could be detected. Ni or other coating material recommended to effectively block the dominant surface diffusion path and improve the reliability. Competitive circuit design should be proposed to minimize the atomic migration for high density circuit in particular.

(3) The increase of the resistance of the daisy chain was not a continuous process, and it often changed significantly in a few minutes or even seconds during the electromigration process due to the rapid propagation of cracks in a very short time.

(4) The failure time was 954.91 h and 588 h based on the atomic flux divergence method and Black’s equation under 1.4 A current and 125 ℃ compared with the experimental result 1065 h based on IPC-9701A guidelines, respectively. The atomic flux divergence method could effectively predict the void formation of solder joints, and life prediction could be more accurate than Black’s equation.

## Figures and Tables

**Table 1:** The material parameters in FEA.

Materials	ρ (g/cm^3^)	E (Gpa)	v	A (1 × 10^−6^/K)
Solder	8.4	30	0.36	24.7
FR4	1.859	11	0.28	13.6
EMC	1.820	8.7	0.3	12.4
BT	1.7	23.1	0.21	12.4
Cu	8.92	117	0.34	16.6

**Table 2:** Packaging structure parameters in FEA.

Component	Parameter	Value (mm)
D101	Length	27
	Width	27
	Height	0.6
D201	Length	15
	Width	15
	Height	0.56
D501	Length	12.3
	Width	11.5
	Height	1

**Table 3:** Parameters of solder joints in each step.

Step	Total Mass Flux Divergence (atoms/m^3^·s)	Average Life of Element (h)
1	1.70 × 10^−4^	3.76
2	1.78 × 10^−4^	3.59
3	1.88 × 10^−4^	3.40
4	2.00 × 10^−4^	3.20
5	2.41 × 10^−4^	2.65
6	2.71 × 10^−4^	2.36
7	2.99 × 10^−4^	2.14
8	3.20 × 10^−4^	2.00
9	3.51 × 10^−4^	1.82
10	4.01 × 10^−4^	1.60

**Table 4:** Failure time results based on different methods.

Method	Failure Time (h)	Error
Experiment	1065 h	/
Black’s equation	588 h	44.78%
AFD	954.91 h	10.33%

## Data Availability

Not applicable.
